# A new humanized *in vitro* model of IgE-mediated rapid desensitization

**DOI:** 10.1186/2045-7022-4-S3-O10

**Published:** 2014-07-18

**Authors:** Matthieu Picard, Joana Caiado, Pedro Giavina-Bianchi, Mariana Castells

**Affiliations:** 1 Brigham and Women's Hospital, Harvard Medical Shcool, Division of Rheumatology, Immunology and Allergy, Massachusetts, USA

## Background

Rapid drug desensitization (RDD) is widely used to re-introduce medications that have caused an IgE-mediated hypersensitivity in allergic patients. However, RDD protocols are largely based on empiric experience and few in vitro models have explored the rationale guiding the rate at which the dose should be increased, what the starting dose should be and how the time elapsed between doses affect the effectiveness of desensitization. This study addresses these issues in a new humanized in vitro model of rapid desensitization.

## Methods

Bone marrow-derived mast cells (BMMCs) from transgenic Balb/c mice expressing the human high affinity IgE receptor alpha chain (hFcRI) were sensitized with human serum containing IgE against dust mites. Sensitized BMMCs were challenged with Dermatophagoides pteronyssinus administered at once (activation) or through a step-wise progressive increase in concentration (desensitization). The influence of the concentration fold-increase per step, of the starting concentration and of the time elapsed between steps on the inhibition of mediator release (-hexosaminidase) induced by desensitization was assessed.

## Results

Inhibition of -hexosaminidase release correlated with the fold-increase per step, the starting concentration and the time elapsed between steps. A two-fold increase per step protocol induced 81% inhibition compared to 51% (p=0.001) and 19% (p=0.003) for four-fold and ten-fold increase per step protocols. Maximal inhibition was reached with a starting concentration below the activation threshold (80%) and a progressive reduction in inhibition was observed with starting concentrations above that threshold. One-minute intervals between steps induced a small degree of inhibition (15%) that was maximal with 10-minute intervals (81%) (p<0.001).

## Conclusion

This *in vitro* humanized mast cell/IgE-mediated desensitization model will allow evaluation of drug hypersensitivity patients and provide the basis for adjusting the fold-increase per step, the starting concentration and the time between steps necessary for safe rapid drug desensitization. These three factors independently influence the inhibition of mediators release and should be taken into account in all RDD protocols.

